# Comparison of seven surrogate insulin resistance indexes for predicting the prevalence of carotid atherosclerosis in normal-weight individuals

**DOI:** 10.3389/fpubh.2023.1241523

**Published:** 2023-08-31

**Authors:** Zeyu Liu, Bi Deng, Qin Huang, Ruxin Tu, Fang Yu, Jian Xia, Jie Feng

**Affiliations:** ^1^Department of Neurology, Xiangya Hospital, Central South University, Changsha, Hunan, China; ^2^Department of Neurology, Peking University People's Hospital, Beijing, China; ^3^Clinical Research Center for Cerebrovascular Disease of Hunan Province, Central South University, Changsha, Hunan, China; ^4^National Clinical Research Center for Geriatric Disorders, Xiangya Hospital, Central South University, Changsha, Hunan, China

**Keywords:** surrogate insulin resistance indexes, carotid atherosclerosis, carotid intima-media thickness, carotid plaque, carotid stenosis, normal-weight individuals

## Abstract

**Introduction:**

The aim of this study was to assess the correlation between surrogate insulin resistance (IR) indexes and carotid atherosclerosis (CA) in normal-weight populations, as well as compared their ability to predict CA.

**Method:**

A total of 26,795 middle-aged and older adult individuals with normal body weights were included. Triglyceride-glucose index (TyG), TyG-body mass index, TyG-waist circumference (TyG-WC), TyG-waist-to-height ratio (TyG-WHtR), visceral adiposity index, Chinese VAI (CVAI) and lipid accumulation product (LAP) were determined using established formulas. The associations between these surrogate indexes and CA were assessed using logistic regression models and restricted cubic spline (RCS) analysis. Receiver operating characteristic curves were utilized to compare the performance of these indexes for predicting CA.

**Result:**

The levels of all seven surrogate indexes of IR were significantly higher in normal-weight individuals with CA than in those without CA (*p* < 0.001). In the full-adjusted model, only CVAI, TyG-WC, TyG-WHtR and LAP were significantly associated with CA, with the adjusted odds ratios (95% CI) of CA being 1.25 (1.20–1.30), 1.18 (1.14–1.23), 1.20 (1.16–1.25) and 1.25 (1.18–1.32) for each one standard deviation increase in CVAI, TyG-WC, TyG-WHtR and LAP, respectively. RCS analysis revealed a significant increase in the prevalence of CA among normal-weight individuals with CVAI >89.83, LAP >28.91, TyG-WHtR >4.42 and TyG-WC >704.93. The area under the curve for CVAI was significantly greater than for other indexes (*p* < 0.001).

**Conclusion:**

CVAI, TyG-WC, TyG-WHtR and LAP were independently associated with the prevalence of CA. Specifically, CVAI may be the most appropriate predictor of CA in normal-weight individuals.

## 1. Introduction

Cardiovascular diseases (CVDs), particularly ischemic heart disease and stroke, remain the leading cause of mortality and a significant contributor to disability globally (1). According to the World Health Organization report from 2019, around 17.9 million deaths were attributed to CVDs, which corresponded to 31% of all the global mortality rate (2). Atherosclerosis, the primary etiology of CVD, poses a significant public health challenge owing to its asymptomatic nature over long terms, unfavorable prognosis, and reduced life expectancy (3, 4). Carotid atherosclerosis (CA), which encompasses increased carotid intima-media thickness (CIMT), plaque and stenosis, is widely recognized as a crucial indicator of generalized atherosclerosis and a predictor of cardiovascular disease events (5–8). Early detection of CA through regular non-invasive ultrasonography is advantageous in implementing proactive measures to prevent or manage CVD before its progression (9). Given the significant and escalating burden of CVD, it is imperative to promptly detect CA in the general population, identify promising biomarkers for early detection and implement preventive measures.

Recent accumulating evidence suggests that obesity, as measured by body mass index (BMI), is strongly associated with an increased risk of developing CA (10, 11). However, a significant number of individuals with normal weight but metabolic abnormalities also exhibit a cluster of metabolic risk factors as well as an elevated risk for carotid artery disease (12, 13). Furthermore, individuals with a normal weight, as determined by BMI, frequently perceive themselves as being in good health. Consequently, it is less probable that individuals with normal weight will undergo clinical screening and early intervention for CA compared to those who are obese. Therefore, timely detection of CA in individuals with normal body weight is imperative.

Insulin resistance (IR), defined as the attenuation of insulin responsiveness in tissues, not only expedites the advancement of atherosclerosis but also serves as a primary characteristic of metabolically obese normal-weight individuals (14). This implies that determining the degree of IR might be advantageous in predicting the likelihood of developing CA. The homeostatic model assessment of insulin resistance (HOMA-IR) has traditionally been employed to quantify insulin resistance; however, there has been no consensus regarding the association of HOMA-IR scores with the risk of CA (15–17). Furthermore, HOMA-IR is substantially limited in clinical practice by the need to measure insulin levels. The demand for a dependable and cost-effective indicator of IR has prompted the creation of innovative surrogate indexes, including triglyceride-glucose index (TyG) (18, 19), TyG-BMI (20), TyG-waist circumference (TyG-WC) (21), TyG-waist-to-height ratio (TyG-WHtR) (22), Chinese visceral adiposity index (CVAI) (23), visceral adiposity index (VAI) (24) and lipid accumulation product (LAP) (18), which are effective in assessing IR status. Reportedly, a few studies have evaluated the association between these partially surrogate IR indexes and atherosclerosis (25–29). However, many of these studies had small sample sizes and did not specifically target individuals with a normal weight, a population that has been understudied and often overlooked in early screening for CA. Furthermore, it remains ambiguous as to which indexes hold greater predictive value for CA.

Despite considerable research on the surrogate indexes of IR, no studies have investigated their relationship with CA prevalence in normal-weight individuals. Accordingly, the present large, cross-sectional study enrolled 26,795 community residents with normal weight to explore the relationship between surrogate IR indexes and CA. In addition, the study identified the index with the most predictive ability for IR in a normal-weight population.

## 2. Materials and methods

### 2.1. Study participants

During the period of 2017–2020, the participants were recruited from the China Stroke High-risk Population Screening and Intervention Program (CSHPSIP) in Hunan province, China (30). The CSHPSIP enrolled community-dwelling adults who met the following criteria: (1) aged >40 years, (2) resided in the community for >6 months, and (3) provided informed consent (31). The Institutional Review Board at Capital Medical University Xuanwu Hospital reviewed and approved the protocol for the CSHPSIP program, and this study was conducted in accordance with the research protocol of CSHPSIP.

**Figure 1:**
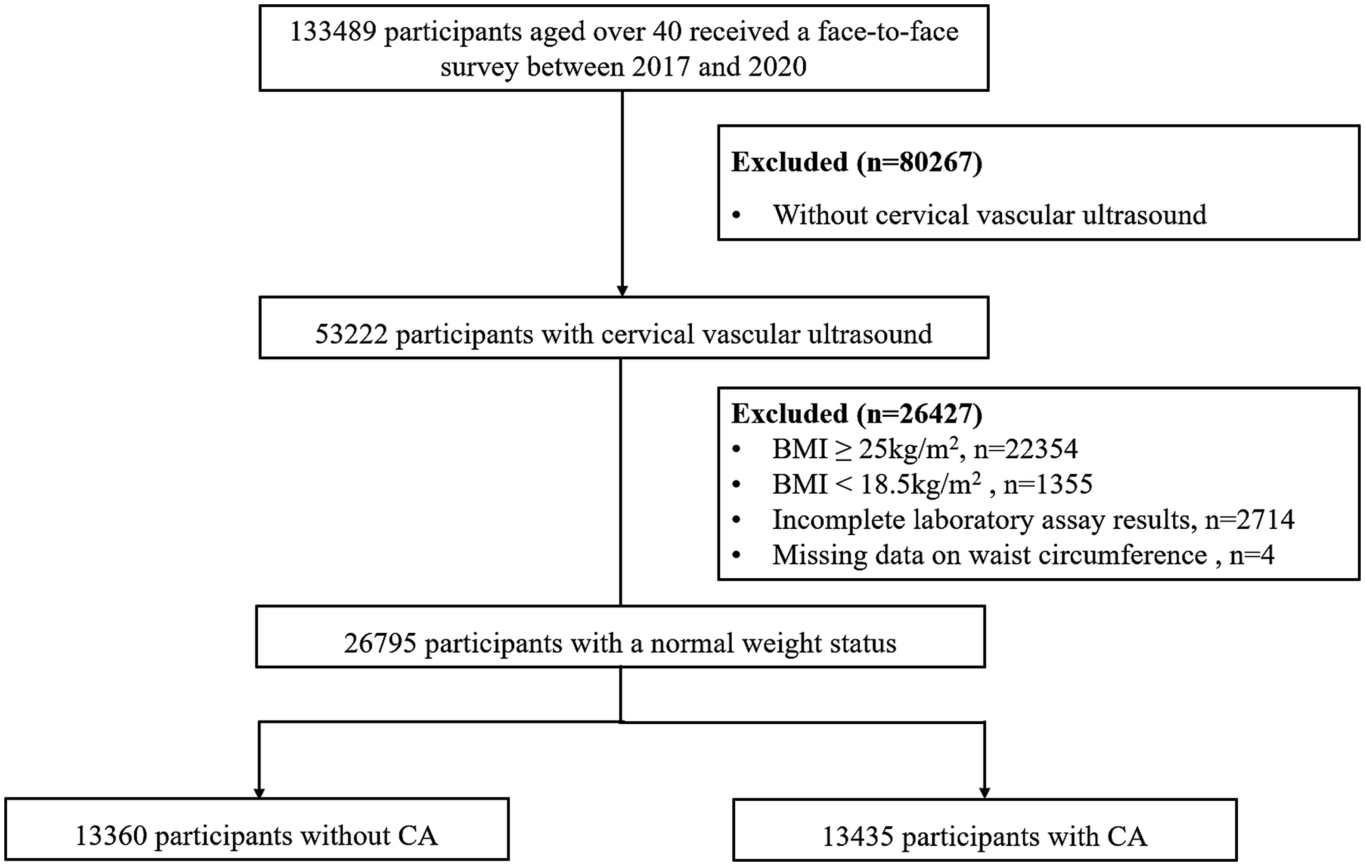
The process of study participants selection.

A total of 26 communities, comprising 13 urban and 13 rural areas, were selected in proportion to local population size and community numbers. Between January 2017 and December 2020, face-to-face surveys were conducted among 133,489 individuals, with 53,222 participants receiving carotid ultrasound examinations in accordance with the screening protocol. The present cross-sectional investigation excluded participants who exhibited abnormal weight, as defined by the World Health Organization standards, including those with a BMI ≥ 25 (*n* = 22,354) or BMI < 18.5 (*n* = 1,355), as well as individuals with incomplete laboratory assay results (*n* = 2,714) and missing data on waist circumference (WC) (*n* = 4). Ultimately, the final analysis included a total of 26,795 normal-weight individuals (Figure 1).

### 2.2. Data collection

A face-to-face interviewer-administered questionnaire was conducted by trained medical staff to gather data on medical, socio-demographic, and lifestyle-related variables. The demographic information collected included age, sex, education level (categorized as “primary school or below,” “middle school,” and “high school or above”), living status, and lifestyle risk factors such as tobacco use, alcohol consumption, and physical activity. Additionally, medical history pertaining to hypertension, diabetes mellitus, cerebrovascular diseases, and heart diseases was obtained. Physical inactivity refers to the absence of moderate-to-vigorous physical activity for >150 min/week or vigorous-intensity physical activity for >75 min/week (31). Diabetes was defined as a fasting plasma glucose level of ≥7.0 mmol/L, a previous diagnosis of diabetes mellitus, or the use of antidiabetic medication or insulin (32). Hypertension was defined as a blood pressure of ≥140/90 mmHg, a history of hypertension, or the use of antihypertensive medication (32). Dyslipidemia was defined as serum total cholesterol (TC) concentration ≥ 6.22 mmol/L, and/or low-density lipoprotein cholesterol (LDL-C) concentration ≥ 4.14 mmol/L, and/or TG concentration ≥ 2.26 mmol/L, and/or HDL-C concentration < 1.04 mmol/L, or previous history of hyperlipidemia (32). The height, weight, and WC were measured twice by a qualified nurse or physician and the results were averaged. BMI was calculated as body mass (in kilograms) divided by the square of height (in meters). Additionally, venous blood samples were collected after an 8 h fast, and laboratory parameters, including fasting blood glucose (FBG), TC, TG, LDL-C, and HDL-C, were analyzed. The seven surrogate indexes of IR were calculated using established formulas, as detailed in Supplementary Table S1.

### 2.3. Definition of carotid atherosclerosis

Two proficient ultrasound technologists, who were unaware of the patient’s clinical information, performed carotid ultrasonography examinations in a skilled and autonomous manner. The participants’ bilateral carotid arteries were scanned in the supine position with the neck in a hyperextended position. The measurement of CIMT was conducted at three distinct locations on the far wall of a 1 cm-long segment of the common carotid artery, situated in close proximity to the carotid bulb. An increased CIMT was defined as a range of 1.0 to 1.5 mm, which is in line with previous research (28). Carotid plaques were identified as having an intima-media thickness exceeding 1.5 mm or protruding into the lumen by 50% more than the surrounding intima-media thickness (28). Carotid stenosis was defined as the occlusion or more than 50% stenosis of at least one common carotid or internal carotid artery (28). Participants exhibiting increased CIMT, plaques, or carotid stenosis were diagnosed with CA.

### 2.4. Statistical analysis

This study utilized counts (proportions) to present categorical variables and medians (interquartile ranges) for non-normally distributed data to present continuous variables. To compare the baseline characteristics of participants without CA to those with CA, the Mann–Whitney test was used for continuous variables and the chi-square test for categorical variables. Three logistic models were employed to evaluate the association between surrogate indexes of IR and CA, such as increased CIMT, carotid plaques, or stenosis. These models consisted of an unadjusted crude model (Model 1), a model adjusted for demographic factors including age, sex, education level, and living status (Model 2), and a model further adjusted for lifestyle factors such as current smoking, alcohol consumption, physical inactivity, medical history of hypertension, diabetes, cerebrovascular disease and heart disease, as well as biochemical markers and anthropometric measurements including FBG, TC, TG, LDL-C, HDL-C, and BMI (Model 3).The variables included in the models all satisfied the criteria of tolerance values greater than 0.1 and variance inflation factor less than 10. *p* for trends was calculated using quartiles of surrogate IR indexes as the ordinal variable. Additionally, surrogate IR indexes were analyzed as continuous variables to investigate the dose–response relationship between a per standard deviation (SD) increase and CA, increased CIMT, carotid plaque, or stenosis. Additional subgroup analyses were conducted to explore the correlation between a per SD increase in CVAI, TyG-WC, TyG-WHtR, or LAP, and CA in diverse subgroups stratified by age (40–49, 50–59, 60–69, and ≥ 70 years), sex (male, female), diabetes (yes, no), and hypertension (yes, no). Additionally, a restricted cubic spline analysis was employed to investigate potential nonlinear associations and visualize the dose–response relationship between surrogate indexes and CA. The study also employed the receiver operator characteristic curve to evaluate the predictive ability of various indexes, including TyG, TyG-BMI, TyG-WC, TyG-WHtR, CVAI, VAI, and LAP, for identifying CA, increased CIMT, carotid plaque, or stenosis. *Z*-tests were utilized to investigate the disparities in area under the curve (AUC) values.

All seven surrogate indexes were calculated based on triglyceride values, thereby raising concerns about potential bias arising from the utilization of anti-dyslipidemia medications. However, medication information for participants in this study was largely missing. Therefore, a sensitivity analysis was conducted by excluding all patients with dyslipidemia from the analyses to assess bias. In the participants without dyslipidemia, we evaluate the predictive capacity of different indexes again and additionally investigate the association between CA and the following measures: TyG-WC, TyG-WHtR, CVAI, and LAP. Detailed results can be found in Supplementary Tables S2, S8.

SPSS version 25.0 (IBM SPSS, Armonk, NY, United States) and R version 4.2.3 (R Development Core Team, Vienna, Austria) were used for all statistical analyses. A two-tailed *p*-value of <0.05 was considered to indicate statistical significance.

## 3. Results

### 3.1. Baseline characteristics

The baseline characteristics of eligible study participants are indicated in Table 1. Of 26,795 normal-weight individuals, 13,435 (50.1%) had CA. The individuals with and without CA had significant differences with regard to age; sex; educational level; living status; smoking and alcohol consumption habits; physical activity levels; history of hypertension, diabetes, cerebrovascular disease and heart disease, as well as baseline serum levels of FBG, TG, TC, LDL-C and HDL-C. Notably, individuals with CA exhibited significantly higher median levels of all surrogate IR indexes (TyG, TyG-BMI, TyG-WC, TyG-WHtR, CVAI, VAI, and LAP) than those without CA.

**Table 1:** Baseline characteristics of study participants with and without CA.

Characteristics	Total (26795)	Non-CA (13360)	CA (13435)	*p* value
Age, years	62 (52–69)	55 (49–65)	66 (58–72)	<0.001
40–49	4,603 (17.2)	3,878 (29.0)	725 (5.4)	<0.001
50–59	7,647 (28.5)	4,612 (34.5)	3,035 (22.6)	
60–69	7,896 (29.5)	2,857 (21.4)	5,039 (37.5)	
≥70	6,649 (24.8)	2013 (15.1)	4,636 (34.5)	
Male, *N* (%)	11,642 (43.4)	5,307 (39.7)	6,335 (47.2)	<0.001
Education, *N* (%)				<0.001
Primary school or below	10,541 (39.3)	4,588 (34.3)	5,953 (44.3)	
Middle school	8,637 (32.2)	4,607 (34.5)	4,030 (30.0)	
High school or above	7,617 (28.4)	4,165 (31.2)	3,452 (25.7)	
Live alone, *N* (%)	1,315 (4.9)	511 (3.8)	804 (6.0)	<0.001
Current smoking, *N* (%)	6,935 (25.9)	2,993 (22.4)	3,942 (29.3)	<0.001
Alcohol consumption, *N* (%)	4,549 (17.0)	2,101 (15.7)	2,448 (18.2)	<0.001
Physical inactivity, *N* (%)	9,971 (37.2)	4,861 (36.4)	5,110 (38.0)	0.003
Hypertension, *N* (%)	13,808 (51.5)	5,419 (40.6)	8,389 (62.4)	<0.001
Diabetes, *N* (%)	7,403 (27.6)	3,078 (23.0)	4,325 (32.2)	<0.001
Cerebrovascular disease, *N* (%)	1,331 (5.0)	318 (2.4)	1,013 (7.5)	<0.001
Heart disease, *N* (%)	1,303 (4.9)	350 (2.6)	953 (7.1)	<0.001
Increased CIMT	10,489 (39.1)		10,489 (78.1)	
Carotid plaques	10,081 (37.6)		10,081 (75.0)	
Carotid stenosis	316 (1.2)		316 (2.4)	
FBG, mmol/L	5.19 (4.50–6.20)	5.10 (4.50–6.00)	5.20 (4.57–6.50)	<0.001
TG, mmol/L	1.50 (1.07–2.16)	1.50 (1.06–2.14)	1.50 (1.08–2.18)	0.027
TC, mmol/L	4.80 (4.10–5.55)	4.78 (4.10–5.49)	4.83 (4.11–5.60)	<0.001
LDL-C, mmol/L	2.60 (2.04–3.25)	2.57 (2.03–3.19)	2.64 (2.05–3.30)	<0.001
HDL-C, mmol/L	1.36 (1.13–1.65)	1.38 (1.14–1.68)	1.35 (1.12–1.62)	<0.001
BMI, kg/m^2^	22.70 (21.30–23.83)	22.72 (21.34–23.83)	22.68 (21.25–23.83)	0.053
WC, cm	80.0 (75.0–85.0)	80.0 (75.0–84.0)	80.0 (76.0–85.0)	<0.001
Surrogate IR indexes				
TyG	8.78 (8.37–9.21)	8.76 (8.35–9.18)	8.79 (8.39–9.24)	<0.001
TyG-BMI	198.48 (183.10–213.32)	198.08 (182.93–212.64)	198.99 (183.26–214.04)	0.005
TyG-WC	702.48 (647.03–761.47)	693.34 (637.77–750.79)	711.44 (655.89–771.18)	<0.001
TyG-WHtR	4.42 (4.07–4.80)	4.35 (4.01–4.71)	4.49 (4.14–4.87)	<0.001
VAI	1.73 (1.09–2.82)	1.71 (1.08–2.75)	1.76 (1.10–2.88)	<0.001
CVAI	88.56 (68.06–109.05)	80.79 (61.15–100.99)	95.99 (76.43–115.30)	<0.001
LAP	27.80 (17.08–44.52)	26.65 (16.40–42.60)	28.80 (17.86–46.25)	<0.001

CA, carotid atherosclerosis; CIMT, carotid intima-media thickness; FBG, fasting blood glucose; TG, triglyceride; TC, total cholesterol; LDL-C, low-density lipoprotein cholesterol; HDL-C, high-density lipoprotein cholesterol; BMI, body mass index; WC, waist circumferences; TyG, triglyceride-glucose index; WHtR, waist-to-height-ratio; VAI, the visceral adiposity index; CVAI, the Chinese visceral adiposity index; LAP, lipid accumulation product.

### 3.2. Association of surrogate IR indexes with CA

**Figure 2:**
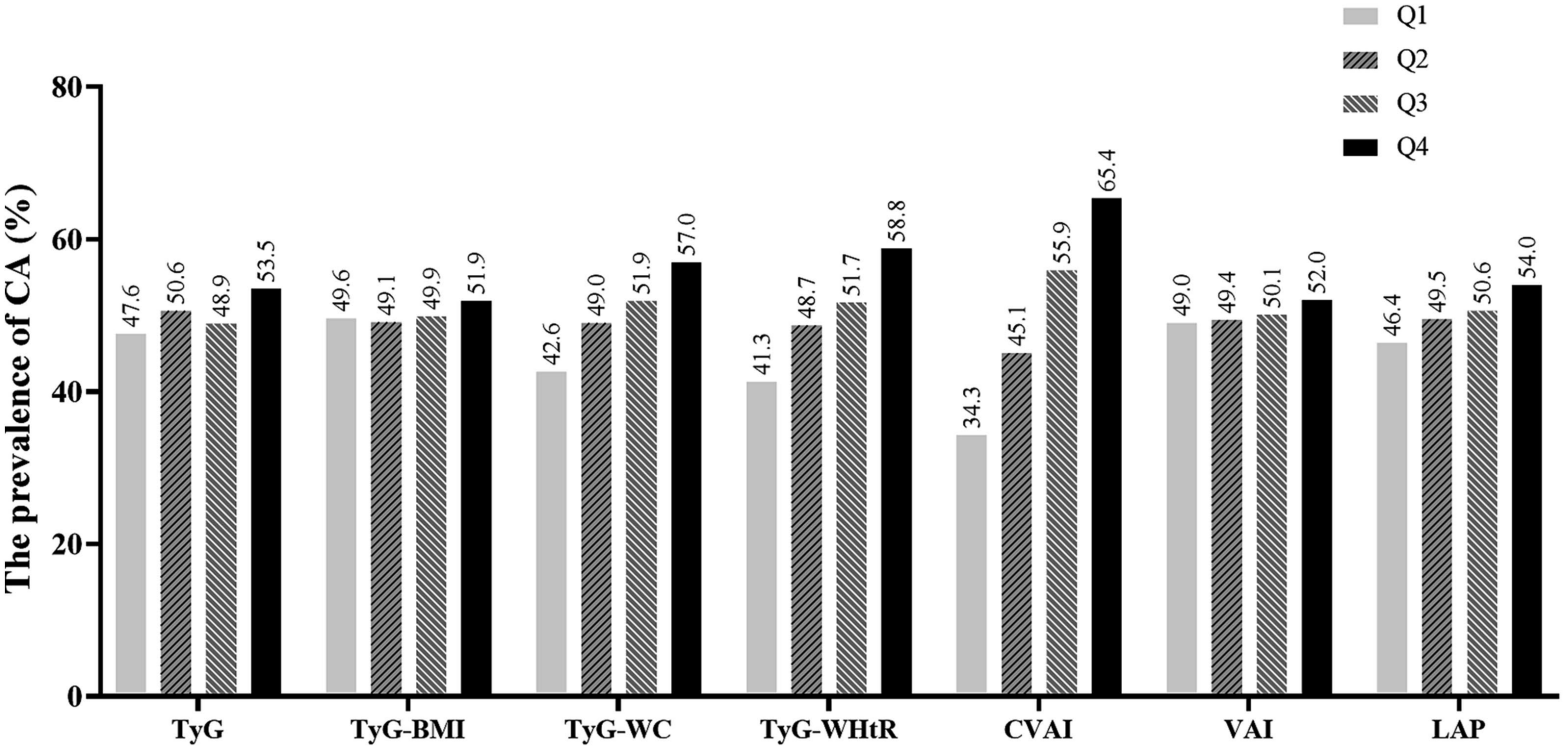
The prevalence of CA for quartiles of seven surrogate IR indexes. CA, carotid atherosclerosis; TyG, triglyceride-glucose index; TyG-BMI, TyG-body mass index; TyG-WC, TyG-waist circumference; TyG-WHtR, TyG-waist-to-height ratio; VAI, the visceral adiposity index; CVAI, the Chinese visceral adiposity index; LAP, lipid accumulation product.

Figure 2 illustrates the relationship between different quartiles of surrogate IR indexes and the prevalence of CA. Briefly, as the levels of surrogate IR indexes increased, the prevalence of CA increased as well (*p* for trend <0.05).

Table 2 shows odds ratios (ORs) and 95% confidence intervals (CIs) of CA by different quartiles of TyG, TyG-BMI, TyG-WC, TyG-WHtR, CVAI, VAI, and LAP. After adjusting for confounding factors, the ORs (95% CIs) for CA as assessed using CVAI were 1.25 (1.15–1.36), 1.47 (1.34–1.61), and 1.69 (1.51–1.88) in quartiles 2, 3 and 4 respectively, compared with those in quartile 1. Similar findings were observed for TyG-WC, TyG-WHtR, and LAP. Furthermore, sensitivity analyses are provided in the Supplementary Table S2, which did not change results above significantly.

**Table 2:** Odds ratio of CA by quartiles of surrogate IR indexes.

Variants	Quartile 1	Quartile 2	Quartile 3	Quartile 4	*p* for trend
TyG					
Model 1	Reference	1.13 (1.06–1.21)	1.05 (0.98–1.13)	1.27 (1.19–1.36)	<0.001
Model 2	Reference	1.07 (0.99–1.15)	0.99 (0.92–1.07)	1.18 (1.09–1.27)	0.001
Model 3	Reference	1.01 (0.94–1.09)	0.89 (0.82–0.97)	1.01 (0.90–1.12)	0.228
TyG-BMI					
Model 1	Reference	0.98 (0.92–1.05)	1.01 (0.95–1.08)	1.10 (1.03–1.17)	0.004
Model 2	Reference	1.02 (0.94–1.10)	1.03 (0.95–1.11)	1.14 (1.05–1.22)	0.001
Model 3	Reference	0.99 (0.90–1.08)	0.97 (0.86–1.08)	1.03 (0.88–1.19)	0.830
TyG-WC					
Model 1	Reference	1.30 (1.21–1.39)	1.45 (1.36–1.55)	1.79 (1.67–1.91)	<0.001
Model 2	Reference	1.15 (1.07–1.24)	1.22 (1.13–1.32)	1.44 (1.34–1.56)	<0.001
Model 3	Reference	1.14 (1.05–1.23)	1.20 (1.11–1.31)	1.44 (1.31–1.58)	<0.001
TyG-WHtR					
Model 1	Reference	1.35 (1.26–1.45)	1.52 (1.42–1.63)	2.03 (1.89–2.17)	<0.001
Model 2	Reference	1.22 (1.13–1.31)	1.26 (1.17–1.35)	1.54 (1.43–1.66)	<0.001
Model 3	Reference	1.21 (1.12–1.31)	1.27 (1.17–1.38)	1.60 (1.45–1.77)	<0.001
CVAI					
Model 1	Reference	1.57 (1.47–1.69)	2.43 (2.27–2.60)	3.62 (3.37–3.89)	<0.001
Model 2	Reference	1.24 (1.15–1.34)	1.45 (1.34–1.57)	1.63 (1.50–1.77)	<0.001
Model 3	Reference	1.25 (1.15–1.36)	1.47 (1.34–1.61)	1.69 (1.51–1.88)	<0.001
VAI					
Model 1	Reference	1.02 (0.95–1.09)	1.05 (0.98–1.12)	1.13 (1.05–1.21)	<0.001
Model 2	Reference	1.10 (1.02–1.18)	1.15 (1.07–1.24)	1.26 (1.17–1.36)	<0.001
Model 3	Reference	1.02 (0.94–1.10)	1.03 (0.94–1.13)	1.13 (1.01–1.27)	0.061
LAP					
Model 1	Reference	1.13 (1.06–1.21)	1.19 (1.11–1.27)	1.36 (1.27–1.45)	<0.001
Model 2	Reference	1.14 (1.06–1.23)	1.17 (1.09–1.26)	1.37 (1.27–1.48)	<0.001
Model 3	Reference	1.13 (1.04–1.22)	1.16 (1.07–1.26)	1.43 (1.30–1.59)	<0.001

Model 1: Unadjusted. Model 2: Adjusted for age, sex, living status, and education level. Model 3: Adjusted for age, sex, living status, education level, current smoking, alcohol consumption, physical inactivity, hypertension, diabetes, cerebrovascular diseases, heart disease, FBG, TC, TG, LDL-C, HDL-C, and BMI.

However, the fully adjusted model revealed significant associations between CA and the fourth quartile of VAI, whereas no such associations were observed for the second and third quartiles. No significant positive correlation was observed between TyG or TyG-BMI and the prevalence of CA among normal-weight individuals. Moreover, the correlation between the surrogate IR indexes and different types of CA suggested that only individuals in the highest quartiles of CVAI or TyG-WHtR exhibited a significantly elevated prevalence for all subtypes of CA (including increased CIMT, plaque and stenosis). Detailed results can be seen in Supplementary Tables S3–S5.

**Figure 3:**
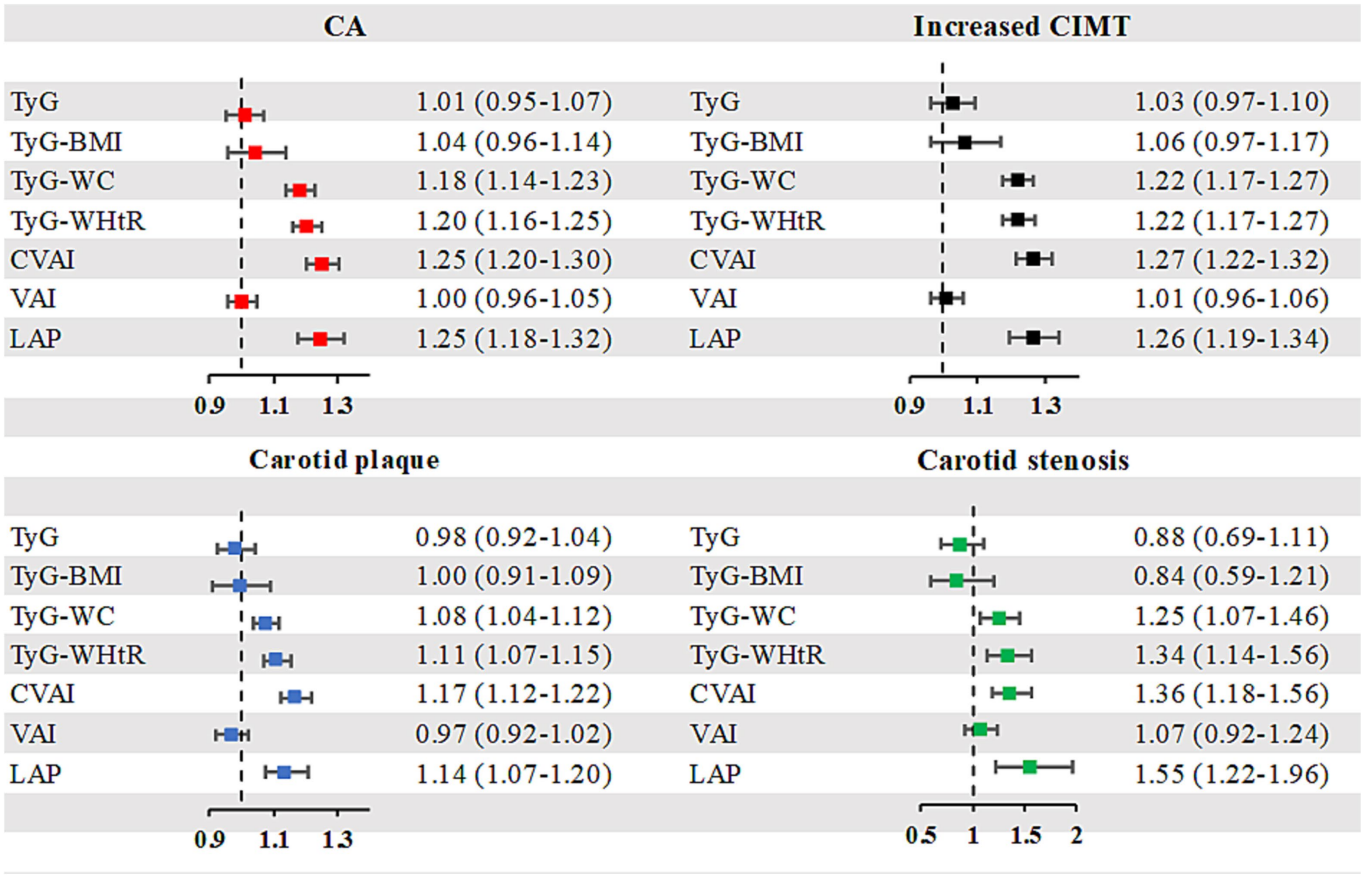
Risk of CA by a per SD increase of surrogate IR indexes. CA, carotid atherosclerosis; CIMT, carotid intima-media thickness; SD, standard deviation; TyG, triglyceride-glucose index; TyG-BMI, TyG-body mass index; TyG-WC, TyG-waist circumference; TyG-WHtR, TyG-waist-to-height ratio; VAI, the visceral adiposity index; CVAI, the Chinese visceral adiposity index; LAP, lipid accumulation product.

The prevalence of CA by per-SD increase of surrogate IR indexes is illustrated in Figure 3. With each additional SD increase in TyG-WC, TyG-WHtR, CVAI, and LAP, the likelihood of CA increased by 18, 20, 25, and 25%, respectively. Similar results were observed for the prevalence of increased CIMT, carotid plaque and carotid stenosis.

**Figure 4:**
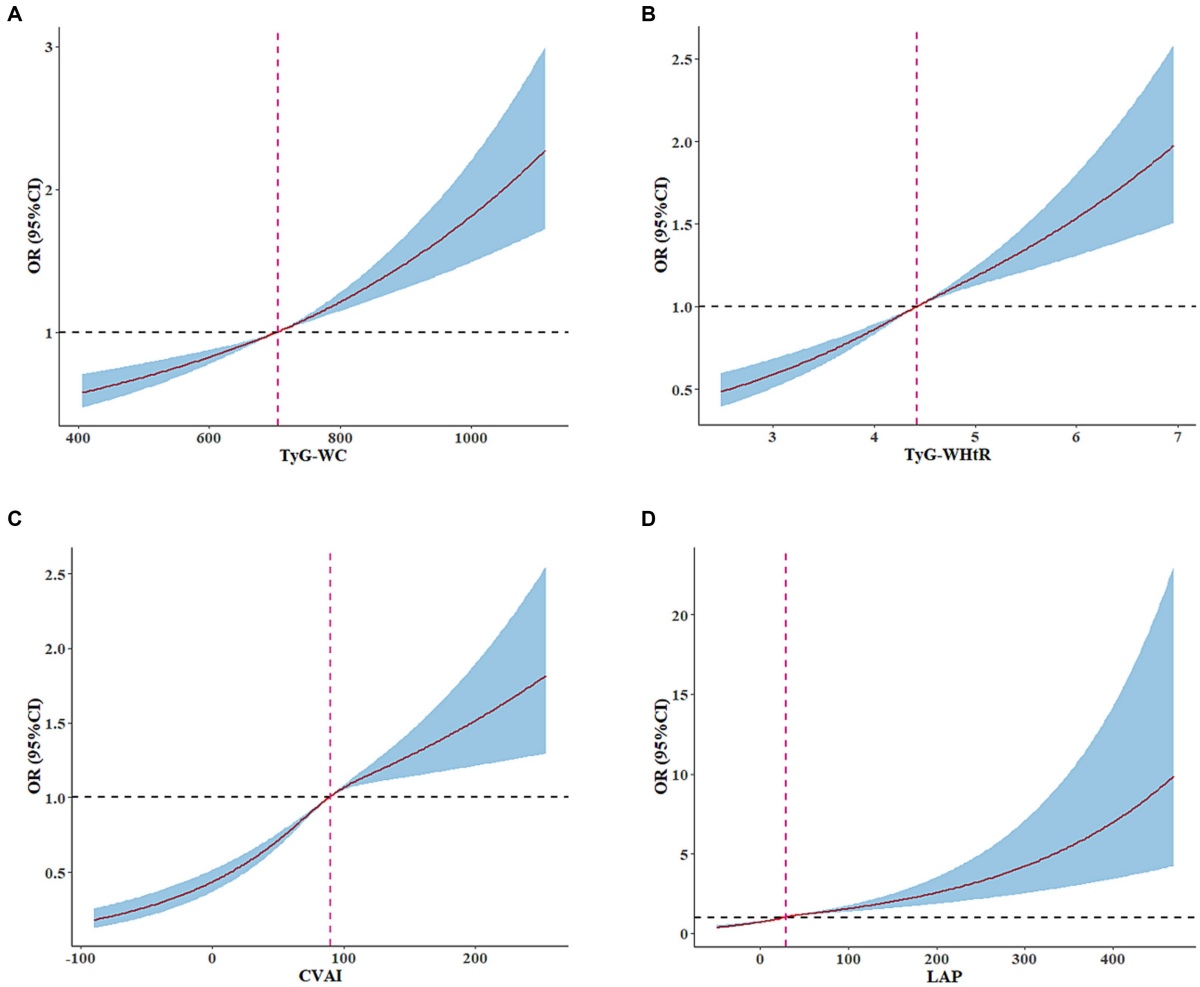
Adjusted cubic spline model of the relationship between TyG-WC, TyG-WHtR, CVAI, LAP, and CA risk. RCS analysis revealed a significant increase in the prevalence of CA among normal-weight individuals with CVAI >89.83, LAP >28.91, TyG-WHtR >4.42 and TyG-WC >704.93. Adjusted for age, sex, living status, education level, current smoking, alcohol consumption, physical inactivity, hypertension, diabetes, cerebrovascular disease, heart disease, FBG, TC, TG, LDL-C, HDL-C, and BMI.

Multivariable restricted cubic spline analysis indicated a significantly elevated rate of CA at higher levels of CVAI, LAP, TyG-WHtR and TyG-WC (Figure 4). Specifically, an appreciable increase was noted in the prevalence of CA among individuals with certain anthropometric indexes such as CVAI >89.83, LAP >28.91, TyG-WHtR >4.42 and TyG-WC >704.93.

### 3.3. Subgroup analyses for the association between surrogate IR indexes and CA prevalence

Table 3 and Supplementary Table S6 display the findings of subgroup analyses that investigate the correlation between CVAI, TyG-WC, TyG-WHtR, and LAP with the prevalence of CA. The relationship between elevated CVAI, TyG-WC, TyG-WHtR, or LAP (per 1 SD) and the prevalence of CA among individuals with normal weight remained consistent across various subgroups. However, a more pronounced association between CVAI, TyG-WC, TyG-WHtR, or LAP and the increased prevalence of CA was observed among females and those aged between 40 and 49 years old.

**Table 3:** Subgroup analyses for the association between a per SD increase of CVAI, TyG-WC, TyG-WHtR, or LAP, and CA.

	CVAI	TyG-WC	TyG-WHtR	LAP
Age, years				
40–49	1.36 (1.19–1.54)	1.28 (1.14–1.43)	1.35 (1.20–1.52)	1.42 (1.18–1.70)
50–59	1.27 (1.18–1.38)	1.21 (1.13–1.29)	1.23 (1.15–1.32)	1.17 (1.07–1.28)
60–69	1.23 (1.14–1.32)	1.15 (1.08–1.23)	1.16 (1.08–1.24)	1.23 (1.12–1.37)
≥70	1.18 (1.09–1.27)	1.14 (1.06–1.23)	1.16 (1.08–1.25)	1.29 (1.15–1.45)
Sex				
Female	1.80 (1.61–2.01)	1.22 (1.16–1.28)	1.21 (1.15–1.28)	1.28 (1.18–1.39)
Male	1.15 (1.10–1.20)	1.13 (1.07–1.19)	1.15 (1.09–1.22)	1.21 (1.12–1.31)
Hypertension				
Yes	1.25 (1.18–1.32)	1.18 (1.13–1.25)	1.19 (1.14–1.26)	1.20 (1.12–1.29)
No	1.23 (1.16–1.31)	1.17 (1.11–1.24)	1.20 (1.14–1.27)	1.33 (1.21–1.46)
Diabetes				
Yes	1.26 (1.16–1.36)	1.18 (1.10–1.26)	1.18 (1.10–1.26)	1.25 (1.14–1.37)
No	1.26 (1.20–1.32)	1.22 (1.16–1.28)	1.25 (1.19–1.31)	1.24 (1.15–1.33)

Adjusted for age, sex, living status, education level, current smoking, alcohol consumption, physical inactivity, hypertension, diabetes, cerebrovascular diseases, heart disease, FBG, TC, TG, LDL-C, HDL-C, and BMI.

### 3.4. Predictive performance of surrogate IR indexes for CA

**Figure 5:**
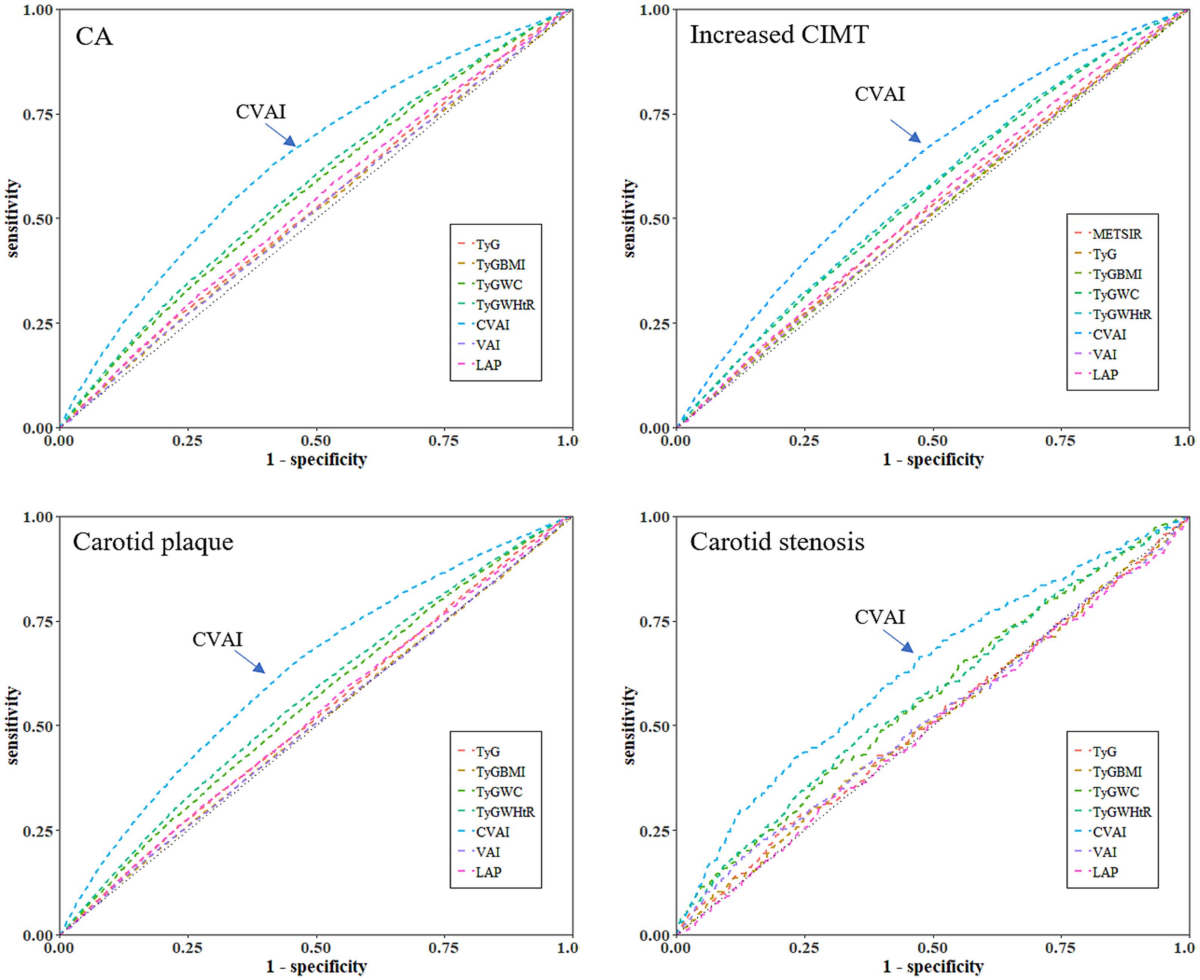
Receiver operating characteristic curves of seven surrogate indexes for predicting CA, increased CIMT, carotid plaque or stenosis. CA, carotid atherosclerosis; CIMT, carotid intima-media thickness; TyG, triglyceride-glucose index; TyG-BMI, TyG-body mass index; TyG-WC, TyG-waist circumference; TyG-WHtR, TyG-waist-to-height ratio; VAI, the visceral adiposity index; CVAI, the Chinese visceral adiposity index; LAP, lipid accumulation product.

Figure 5 and Supplementary Table S7 demonstrate the predictive performance of seven surrogate indexes for CA. CVAI exhibited the largest AUC of 0.638 (*p* < 0.001). Moreover, the optimal CVAI value for detecting CA in normal-weight individuals was 86.69, with a sensitivity of 0.631 and specificity of 0.583. Furthermore, CVAI exhibited superior accuracy in predicting all assessed subtypes of CA, including increased CIMT, carotid plaque and carotid stenosis (all *p* < 0.001). The results of the sensitivity analyses, which did not significantly change the results, are provided in the Supplementary Table S8.

## 4. Discussion

Using data from CSHPSIP, this large-scale, cross-sectional study demonstrated a significant correlation between the prevalence of CA and TyG-WC, TyG-WHtR, CVAI, and LAP in normal-weight adults. However, no significant association was observed between the prevalence of CA and TyG, TyG-BMI or VAI. Furthermore, among all the indexes, CVAI exhibited superior predictive ability for determining CA, increased CIMT, plaque and stenosis.

Research suggests that atherosclerosis is characterized by an initial, extended asymptomatic phase, which can commence as early as adolescence or even childhood (33). Given the increasing burden of CVD, it is crucial to conduct early screening and timely interventions for atherosclerosis for reducing the incidence of CVD. Furthermore, it is less likely that the normal-weight population will undergo clinical screening and early intervention for CA compared with the obese population. However, the present study found that the prevalence of CA was as high as 50.1% in normal-weight individuals. This indicates that it is imperative to incorporate carotid ultrasound into routine annual physical examinations even for individuals with normal body weight.

IR is a robust predictor of atherosclerotic CVD, as it plays a pivotal role in the initiation and progression of atherosclerosis via complex pathophysiological processes such as endothelial injury, activation of the inflammatory response and oxidative stress (34). Many observational studies have shown that over 30% of individuals with a normal body weight exhibit metabolic abnormalities, such as abdominal adiposity and insulin resistance, similar to those typically seen in overweight or obese individuals (known as the “MONW” phenotype) (35, 36). Furthermore, Asian populations who are metabolically obese but have normal weight are at an elevated risk of developing CA compared with metabolically normal but obese or normal-weight individuals (12). However, there is a dearth of metabolic indicators to determine the onset of CA in individuals with normal body weight. To the best of our knowledge, the present study is the first to enroll 26,795 normal-weight individuals and to demonstrate that CVAI is the most effective surrogate index for predicting CA. Moreover, CVAI can be used as a reliable and easily quantifiable measure to identify high-risk populations and implement primary prevention strategies against atherosclerosis.

CVAI is indicative of the distribution of abdominal fat and dyslipidemia and is linked to insulin resistance, abnormal glucose metabolism and an elevated risk of CVD in adults (23, 37, 38). In a cohort study of 3,640 Chinese individuals from CHARLS, CVAI exhibited a higher predictive ability for metabolic syndrome in females than the other six surrogate indexes examined in our study (39). Another study enrolling 1,452 Chinese adults similarly demonstrated that CVAI had a superior predictive ability for identifying metabolic syndrome (40). In certain studies, the correlation between CVAI and CA has also been examined. A study based on 4,075 workers from a steel company has revealed that an increased CVAI is a predictive indicator of increased CIMT (29). However, it should be noted that steelworkers are more overweight and obese than the general population owing to occupational factors such as shift work, noise exposure and occupational stress (29). As such, the findings may not be generalizable to individuals within the normal-weight range. Furthermore, a retrospective study in Taiwan Province revealed a positive correlation between CVAI and the prevalence of CA (41). In addition, the study by Hu et al. observed a significant association between CVAI and the risk of carotid plaque (42). However, these studies did not include participants with normal weight; thus, the results may not be generalizable to the Chinese population with normal weight. Compared with previous studies, the present study specifically examined individuals with normal weight and identified a non-linear, positive association between CVAI and CA, thereby addressing gaps in our understanding of the relationship between CVAI and CA occurrence among normal-weight individuals. Notably, when CVAI exceeds 89.83, the prevalence of CA increases significantly. Therefore, for adults with normal weight, it is recommended to undergo further carotid ultrasound examination when their CVAI exceeds 89.83.

The LAP index, a measure of obesity that is based on WC and fasting TG level, is used for determining the burden of coronary atherosclerotic plaques and the risk of CVD (43, 44). To date, only one study has indicated a correlation between LAP and carotid plaque; however, its small sample size and exclusive focus on acromegalic populations limits the generalizability of the findings (27). The present study revealed a significant correlation between an increase of one SD in LAP and the development of atherosclerotic phenotype, particularly stenosis, among individuals with normal BMI. However, further research is necessary to validate the applicability of these findings to individuals who are either obese or underweight.

The TyG index serves as a practical surrogate for IR and exhibits superior performance compared with the traditional HOMA-IR (45). However, there has been no consensus regarding the correlation between elevated TyG and an increased prevalence of CA. In respective studies, Irace et al. and Li et al. demonstrated a significant correlation between the TyG index and CA (15, 46). However, Zhao et al. failed to establish any association between the TyG index and increased CIMT or carotid plaque (47). Irrespective, detailed analyses of different types of CA or subgroups of participants with normal weight were not conducted in these studies. Li et al. revealed that overweight individuals in the fourth quartile of TyG were at a higher risk for developing CA, increased CIMT, plaque formation and stenosis than those in the first quartile (28). Nevertheless, there were no notable correlations between either carotid plaques or stenosis with regard to individuals with normal BMI in the second to fourth quarters of the TyG index (28). The current investigation additionally revealed no significant correlation of TyG with CA, increased CIMT, plaque, and stenosis. These findings suggest that TyG may not be a dependable indicator for evaluating CA, particularly in individuals with normal BMI.

TyG index-related parameters are composite indicators that incorporate the TyG index with BMI, WC, and WHtR, as initially proposed by Er et al. (20). As mounting evidence suggests a close association between visceral adiposity and IR, the combination of visceral adiposity and TyG may offer a greater potential for identifying IR than TyG alone (20–22). Similarly, although the current study revealed no significant correlation between TyG and increased prevalence of CA among normal-weight participants, a noteworthy association was observed when combined with a composite measure of visceral adiposity (including WC and WHtR).

The association between VAI and atherosclerosis has yielded inconsistent findings in prior research studies. Several studies, despite their relatively small sample sizes, demonstrated a significant correlation between VAI and CA in the general population (25, 26). Xu et al. enrolled 3,363 older adult Chinese individuals and demonstrated that increased VAI was not linked with CA risk (48). Similarly, a cross-sectional study involving 788 Spanish patients did not reveal any correlation between VAI and CA risk (49).The sample size in this study was higher than that reported previously, leading to more robust findings. However, there was no significant correlation between an increase in per VAI SD and an elevated prevalence of CA among individuals with normal weight. This suggests that VAI may not be an effective predictor of CA.

The subgroup analysis conducted in this study demonstrated that the impact of CVAI, TyG-WC, TyG-WHtR, and LAP on the prevalence of CA was notably more significant among females and individuals aged between 40 and 49 years. This finding may be attributed to the heightened risk of atherosclerotic complications during the menopausal transition period for females, as previously suggested in literature (50). The decrease in estrogen secretion during peri-menopause and menopause is known to result in the accumulation of central adiposity and insulin resistance (51). In the context of insulin resistance, the absence of estrogen’s safeguarding impact on endothelial function increases the vulnerability to atherosclerosis. To substantiate this conjecture, additional external validation in more representative populations is imperative.

While benefiting from the well-established cohort and its relatively large size, this study is subject to certain limitations. First, certain confounding factors such as dietary habits and postmenopausal status were not considered in this investigation, which may have affected study results. Second, the study could not determine causality between surrogate IR indexes and CA due to its cross-sectional design. Finally, the present findings should be cautiously generalized to other populations, as the study only included Chinese middle-aged and older adult participants with normal weight.

## 5. Conclusion

Our study has provided evidence suggesting that CVAI, TyG-WC, TyG-WHtR, and LAP are potential predictors of CA in middle-aged and older adult individuals who have normal weight. Specifically, CVAI may be the most appropriate index for predicting CA in a normal-weight population.

## Data availability statement

The raw data supporting the conclusions of this article will be made available by the authors, without undue reservation.

## Ethics statement

The studies involving humans were reviewed and approved by Xiangya Hospital Ethics Committee. The protocol and informed consent for the study of the China Stroke High-risk Population Screening and Intervention Program were reviewed and approved by the Institutional Review Board at the Capital Medical University Xuanwu Hospital early. The studies were conducted in accordance with the local legislation and institutional requirements. The participants provided their written informed consent to participate in this study.

## Author contributions

ZL and JX: conceptualization. JF: data curation, resources, and writing – review and editing. ZL: formal analysis and writing – original draft. RT: investigation. ZL, BD, and QH: methodology. JX: project administration. BD, QH, and FY: software. RT, FY, and JF: validation. All authors contributed to the article and approved the submitted version.

## Funding

This research was supported by the National Key Research and Development Projects (2022YFC3602400, 2022YFC3602401), the National Natural Science Foundation of China (82271369), the National Natural Science Foundation Youth Fund (82001392), the Natural Science Foundation of Hunan Province (Grant No. 2021JJ31109), and the Fundamental Research Funds for the Central Universities of Central South University (2022ZZTS0821).

## Conflict of interest

The authors declare that the research was conducted in the absence of any commercial or financial relationships that could be construed as a potential conflict of interest.

## Publisher’s note

All claims expressed in this article are solely those of the authors and do not necessarily represent those of their affiliated organizations, or those of the publisher, the editors and the reviewers. Any product that may be evaluated in this article, or claim that may be made by its manufacturer, is not guaranteed or endorsed by the publisher.
