# Supplementary material for: Comparison of seven surrogate insulin resistance indexes for predicting the prevalence of carotid atherosclerosis in normal-weight individuals
Source: Front Public Health. 2023 Aug 31;11:1241523. doi: 10.3389/fpubh.2023.1241523 (PMC10501451; doi:10.3389/fpubh.2023.1241523)
Supplement: Supplementary file 1 [file Table_1.DOCX]

Supplementary Material

Elevated Chinese visceral adiposity index increases the risk of stroke in Chinese patients with metabolic syndrome

**Zeyu Liu, Bi Deng, Qin Huang, Ruxin Tu, Fang Yu, Jian Xia, Jie Feng***

*** Correspondence:** Jie Feng: 4010009@csu.edu.cn.

| **Supplement Table 1. Assessment of seven surrogate IR indexes** | |
| --- | --- |
| **TyG** | Ln (fasting TG [mg/dL] × FBG [mg/dL] / 2) |
| **TyG-BMI** | TyG × BMI (kg/m^2^) |
| **TyG-WC** | TyG × WC (cm) |
| **TyG-WHtR** | TyG × (WC [cm] / Height [cm]) |
| **CVAI (Male)** | −267.93 + 0.68 × age (years) + 0.03 × BMI (kg/m^2^) + 4.00 × WC (cm) + 22.00 × log_10_(TG [mmol/L]) − 16.32 × HDL-C (mmol/L) |
| **CVAI (Female)** | −187.32 + 1.71 × age (years) + 4.32 × BMI (kg/m^2^) + 1.12 × WC (cm) + 39.76 × log_10_(TG [mmol/L]) − 11.66 × HDL-C (mmol/L) |
| **VAI (Male)** | WC (cm) / (39.68+(1.88 × BMI [kg/m^2^])) × (TG [mmol/L] / 1.03) × (1.31 / HDL-C [mmol/L]) |
| **VAI (Female)** | WC (cm) / (36.58+(1.89 × BMI [kg/m^2^])) × (TG [mmol/L] / 0.81) × (1.52 / HDL-C [mmol/L]) |
| **LAP (Male)** | LAP = (WC [cm] – 65) × TG (mmol/L) |
| **LAP (Female)** | LAP = (WC [cm] – 58) × TG (mmol/L) |

Abbreviations: IR, insulin resistance; FBG, fasting blood glucose; TG, triglyceride; HDL-C, high-density lipoprotein cholesterol; BMI, body mass index; WC, waist circumferences; TyG, triglyceride‐glucose index; WHtR, waist-to-height-ratio; VAI, the visceral adiposity index; CVAI, the Chinese visceral adiposity index; LAP, lipid accumulation product.

| **Supplement Table 2. Odds ratio of CA by quartiles of TyG-WC, TyG-WHtR, CVAI and LAP among individuals** **without dyslipidemia.** | | | | | |
| --- | --- | --- | --- | --- | --- |
| **Variants** | **Quartile 1** | **Quartile 2** | **Quartile 3** | **Quartile 4** | ***P* for trend** |
| **TyG-WC** | | | | | |
| Model 1 | Reference | 1.30 (1.18-1.43) | 1.36 (1.24-1.49) | 1.75 (1.59-1.92) | < 0.001 |
| Model 2 | Reference | 1.17 (1.06-1.31) | 1.08 (0.97-1.32) | 1.27 (1.14-1.41) | < 0.001 |
| Model 3 | Reference | 1.18 (1.05-1.32) | 1.11 (0.98-1.25) | 1.31 (1.14-1.51) | 0.002 |
| **TyG-WHtR** | | | | | |
| Model 1 | Reference | 1.28 (1.16-1.41) | 1.55 (1.41-1.70) | 2.04 (1.85-2.24) | <0.001 |
| Model 2 | Reference | 1.14 (1.02-1.26) | 1.16 (1.05-1.29) | 1.36 (1.22-1.51) | <0.001 |
| Model 3 | Reference | 1.17 (1.05-1.31) | 1.25 (1.10-1.41) | 1.54 (1.33-1.77) | <0.001 |
| **CVAI** | | | | | |
| Model 1 | Reference | 1.60 (1.45-1.77) | 2.54 (2.30-2.80) | 4.27 (3.86-4.72) | <0.001 |
| Model 2 | Reference | 1.25 (1.12-1.40) | 1.40 (1.25-1.57) | 1.57 (1.39-1.77) | <0.001 |
| Model 3 | Reference | 1.27 (1.13-1.43) | 1.42 (1.25-1.62) | 1.60 (1.37-1.87) | <0.001 |
| **LAP** | | | | | |
| Model 1 | Reference | 1.09 (1.00-1.20) | 1.11 (1.01-1.22) | 1.24 (1.13-1.36) | <0.001 |
| Model 2 | Reference | 1.13 (1.01-1.25) | 1.15 (1.03-1.28) | 1.23 (1.11-1.38) | <0.001 |
| Model 3 | Reference | 1.17 (1.05-1.31) | 1.23 (1.08-1.40) | 1.48 (1.26-1.73) | <0.001 |
| **Model 1:** Unadjusted. **Model 2:** Adjusted for age, sex, living status, education level. **Model 3:** Adjusted for age, sex, living status, education level, current smoking, alcohol consumption, physical inactivity, hypertension, diabetes, cerebrovascular diseases, heart disease, FBG, TC, TG, LDL-C, HDL-C, and BMI. | | | | | |

| **Supplement Table 3. Odds ratio of increased CIMT by quartiles of surrogate IR indexes.** | | | | | |
| --- | --- | --- | --- | --- | --- |
| **Variants** | **Quartile 1** | **Quartile 2** | **Quartile 3** | **Quartile 4** | ***P* for trend** |
| **TyG** | | | | | |
| Model 1 | Reference | 1.05(0.98-1.13) | 1.00(0.93-1.07) | 1.15(1.07-1.23) | 0.001 |
| Model 2 | Reference | 1.00(0.93-1.08) | 0.96(0.89-1.03) | 1.08(1.00-1.16) | 0.105 |
| Model 3 | Reference | 0.97(0.90-1.05) | 0.91(0.84-1.00) | 1.04 (0.93-1.17) | 0.663 |
| **TyG-BMI** | | | | | |
| Model 1 | Reference | 0.99(0.93-1.07) | 1.03(0.96-1.11) | 1.08(1.00-1.15) | 0.021 |
| Model 2 | Reference | 1.02(0.94-1.09) | 1.04(0.96-1.12) | 1.09(1.02-1.18) | 0.015 |
| Model 3 | Reference | 0.99(0.90-1.09) | 0.99(0.88-1.11) | 1.05(0.90-1.23) | 0.526 |
| **TyG-WC** | | | | | |
| Model 1 | Reference | 1.36(1.26-1.46) | 1.48(1.38-1.58) | 1.73(1.61-1.86) | < 0.001 |
| Model 2 | Reference | 1.23(1.14-1.33) | 1.27(1.18-1.37) | 1.43(1.33-1.54) | < 0.001 |
| Model 3 | Reference | 1.25(1.15-1.35) | 1.30(1.19-1.41) | 1.54(1.40-1.70) | < 0.001 |
| **TyG-WHtR** | | | | | |
| Model 1 | Reference | 1.39(1.30-1.50) | 1.51(1.41-1.62) | 1.84(1.72-1.98) | < 0.001 |
| Model 2 | Reference | 1.28(1.19-1.38) | 1.29(1.20-1.40) | 1.49(1.38-1.60) | < 0.001 |
| Model 3 | Reference | 1.29(1.19-1.40) | 1.35(1.24-1.47) | 1.67(1.50-1.83) | < 0.001 |
| **CVAI** | | | | | |
| Model 1 | Reference | 1.60(1.49-1.73) | 2.25(2.09-2.42) | 3.10(2.88-3.33) | < 0.001 |
| Model 2 | Reference | 1.32(1.22-1.42) | 1.48(1.37-1.60) | 1.66(1.53-1.81) | < 0.001 |
| Model 3 | Reference | 1.34(1.23-1.46) | 1.54(1.40-1.68) | 1.81(1.63-2.01) | < 0.001 |
| **VAI** | | | | | |
| Model 1 | Reference | 1.02(0.96-1.10) | 1.04(0.97-1.12) | 1.12(1.04-1.20) | 0.002 |
| Model 2 | Reference | 1.09(1.01-1.17) | 1.12(1.04-1.21) | 1.23(1.14-1.32) | < 0.001 |
| Model 3 | Reference | 1.04(0.96-1.13) | 1.08(0.98-1.18) | 1.27(1.12-1.43) | 0.001 |
| **LAP** | | | | | |
| Model 1 | Reference | 1.19(1.11-1.28) | 1.22(1.14-1.31) | 1.34(1.25-1.43) | <0.001 |
| Model 2 | Reference | 1.20(1.11-1.29) | 1.20(1.12-1.30) | 1.34(1.24-1.44) | <0.001 |
| Model 3 | Reference | 1.21(1.11-1.30) | 1.25(1.15-1.36) | 1.55(1.40-1.72) | <0.001 |
| **Model 1:** Unadjusted. **Model 2:** Adjusted for age, sex, living status, education level. **Model 3:** Adjusted for age, sex, living status, education level, current smoking, alcohol consumption, physical inactivity, hypertension, diabetes, cerebrovascular diseases, heart disease, FBG, TC, TG, LDL-C, HDL-C, and BMI. | | | | | |

| **Supplement Table 4. Odds ratio of carotid plaques by quartiles of surrogate IR indexes.** | | | | | |
| --- | --- | --- | --- | --- | --- |
| **Variants** | **Quartile 1** | **Quartile 2** | **Quartile 3** | **Quartile 4** | ***P* for trend** |
| **TyG** | | | | | |
| Model 1 | Reference | 1.12(1.04-1.20) | 1.05(0.98-1.13) | 1.23(1.14-1.32) | < 0.001 |
| Model 2 | Reference | 1.07(0.99-1.15) | 1.00(0.92-1.07) | 1.15(1.07-1.24) | 0.004 |
| Model 3 | Reference | 1.00(0.92-1.08) | 0.88(0.81-0.96) | 0.94(0.84-1.05) | 0.033 |
| **TyG-BMI** | | | | | |
| Model 1 | Reference | 0.94(0.88-1.01) | 0.97(0.90-1.04) | 1.02(0.95-1.10) | 0.427 |
| Model 2 | Reference | 0.99(0.92-1.07) | 1.00(0.93-1.08) | 1.08(1.00-1.16) | 0.057 |
| Model 3 | Reference | 0.95(0.86-1.04) | 0.92(0.82-1.03) | 0.93(0.80-1.09) | 0.361 |
| **TyG-WC** | | | | | |
| Model 1 | Reference | 1.23(1.15-1.32) | 1.34(1.25-1.44) | 1.57(1.47-1.69) | < 0.001 |
| Model 2 | Reference | 1.09(1.01-1.17) | 1.12(1.04-1.21) | 1.27(1.17-1.37) | < 0.001 |
| Model 3 | Reference | 1.06(0.98-1.15) | 1.08(0.99-1.17) | 1.18(1.07-1.31) | 0.001 |
| **TyG-WHtR** | | | | | |
| Model 1 | Reference | 1.23(1.15-1.33) | 1.42(1.32-1.52) | 1.79(1.67-1.92) | < 0.001 |
| Model 2 | Reference | 1.11(1.03-1.20) | 1.17(1.08-1.26) | 1.35(1.25-1.46) | < 0.001 |
| Model 3 | Reference | 1.09(1.00-1.18) | 1.15(1.06-1.26) | 1.32(1.20-1.46) | < 0.001 |
| **CVAI** | | | | | |
| Model 1 | Reference | 1.47(1.36-1.58) | 2.15(2.00-2.32) | 3.20(2.97-3.44) | < 0.001 |
| Model 2 | Reference | 1.17(1.08-1.27) | 1.30(1.20-1.42) | 1.42(1.30-1.55) | < 0.001 |
| Model 3 | Reference | 1.17(1.07-1.27) | 1.30(1.18-1.42) | 1.43(1.28-1.60) | < 0.001 |
| **VAI** | | | | | |
| Model 1 | Reference | 0.96 (0.89-1.03) | 0.98(0.92-1.05) | 1.02(0.95-1.09) | 0.455 |
| Model 2 | Reference | 1.04 (0.96-1.12) | 1.09(1.01-1.18) | 1.16(1.07-1.25) | < 0.001 |
| Model 3 | Reference | 0.95 (0.87-1.03) | 0.95(0.87-1.05) | 0.97(0.86-1.09) | 0.580 |
| **LAP** | | | | | |
| Model 1 | Reference | 1.05 (0.98-1.13) | 1.06(0.99-1.14) | 1.19(1.11-1.28) | < 0.001 |
| Model 2 | Reference | 1.07 (0.99-1.15) | 1.06(0.98-1.15) | 1.21(1.12-1.31) | < 0.001 |
| Model 3 | Reference | 1.04 (0.96-1.13) | 1.03(0.95-1.12) | 1.18(1.07-1.31) | 0.006 |
| **Model 1:** Unadjusted. **Model 2:** Adjusted for age, sex, living status, education level. **Model 3:** Adjusted for age, sex, living status, education level, current smoking, alcohol consumption, physical inactivity, hypertension, diabetes, cerebrovascular diseases, heart disease, FBG, TC, TG, LDL-C, HDL-C, and BMI. | | | | | |

| **Supplement Table 5. Odds ratio of carotid stenosis by quartiles of surrogate IR indexes.** | | | | | |
| --- | --- | --- | --- | --- | --- |
| **Variants** | **Quartile 1** | **Quartile 2** | **Quartile 3** | **Quartile 4** | ***P* for trend** |
| **TyG** | | | | | |
| Model 1 | Reference | 0.97(0.71-1.32) | 0.85(0.61-1.18) | 1.17(0.86-1.8) | 0.447 |
| Model 2 | Reference | 0.97(0.70-1.33) | 0.87(0.63-1.21) | 1.21(0.89-1.64) | 0.310 |
| Model 3 | Reference | 0.87(0.63-1.21) | 0.73(0.51-1.06) | 0.91(0.57-1.45) | 0.373 |
| **TyG-BMI** | | | | | |
| Model 1 | Reference | 0.84(0.62-1.15) | 0.79(0.58-1.09) | 0.95(0.71-1.29) | 0.685 |
| Model 2 | Reference | 0.90(0.66-1.24) | 0.86(0.62-1.17) | 1.08(0.80-1.46) | 0.750 |
| Model 3 | Reference | 0.78(0.53-1.14) | 0.67(0.41-1.08) | 0.72(0.38-1.35) | 0.288 |
| **TyG-WC** | | | | | |
| Model 1 | Reference | 1.31(0.93-1.84) | 1.38(0.98-1.93) | 1.69(1.22-2.34) | 0.002 |
| Model 2 | Reference | 1.15(0.82-1.62) | 1.14(0.81-1.60) | 1.33(0.96-1.84) | 0.103 |
| Model 3 | Reference | 1.11(0.78-1.58) | 1.07(0.74-1.54) | 1.19(0.79-1.79) | 0.494 |
| **TyG-WHtR** | | | | | |
| Model 1 | Reference | 1.38(0.98-1.95) | 1.31(0.93-1.86) | 1.97(1.42-2.72) | < 0.001 |
| Model 2 | Reference | 1.29(0.91-1.82) | 1.16(0.82-1.65) | 1.70(1.22-2.37) | 0.004 |
| Model 3 | Reference | 1.31(0.92-1.88) | 1.20(0.82-1.76) | 1.79(1.18-2.72) | 0.016 |
| **CVAI** | | | | | |
| Model 1 | Reference | 1.47(1.36-1.58) | 2.15(2.00-2.32) | 3.20(2.97-3.44) | < 0.001 |
| Model 2 | Reference | 0.92(0.67-1.47) | 1.13(0.78-1.65) | 1.60(1.12-2.28) | 0.002 |
| Model 3 | Reference | 1.04(0.69-1.55) | 1.23(0.82-1.84) | 1.81(1.18-2.79) | 0.002 |
| **VAI** | | | | | |
| Model 1 | Reference | 0.84(0.62-1.15) | 0.82(0.60-1.12) | 0.89(0.65-1.20) | 0.418 |
| Model 2 | Reference | 1.00(0.73-1.37) | 1.06(0.77-1.45) | 1.25(0.91-1.73) | 0.175 |
| Model 3 | Reference | 0.92(0.65-1.31) | 0.93(0.62-1.40) | 1.11(0.64-1.93) | 0.830 |
| **LAP** | | | | | |
| Model 1 | Reference | 0.97(0.71-1.33) | 0.91(0.67-1.25) | 1.01(0.74-1.38) | 0.958 |
| Model 2 | Reference | 1.07(0.78-1.47) | 1.05(0.76-1.45) | 1.24(0.90-1.70) | 0.239 |
| Model 3 | Reference | 1.03 (0.75-1.43) | 1.02(0.71-1.46) | 1.20(0.78-1.87) | 0.496 |
| **Model 1:** Unadjusted. **Model 2:** Adjusted for age, sex, living status, education level. **Model 3:** Adjusted for age, sex, living status, education level, current smoking, alcohol consumption, physical inactivity, hypertension, diabetes, cerebrovascular diseases, heart disease, FBG, TC, TG, LDL-C, HDL-C, and BMI. | | | | | |

| **Supplement Table 6. Subgroup analyses for the association between a per SD increase of CVAI, TyG-WC, TyG-WHtR, or LAP with increased CIMT, carotid plaques, and carotid stenosis.** | | | | | |
| --- | --- | --- | --- | --- | --- |
|  |  |  | **CIMT** | **Plaques** | **Stenosis** |
| **CVAI** | **Age** | 40-49 | 1.33(1.16-1.52) | 1.27(1.09-1.48) | 2.13(0.34-13.47) |
|  |  | 50-59 | 1.27(1.17-1.38) | 1.20(1.10-1.31) | 1.78(1.12-2.83) |
|  |  | 60-69 | 1.27(1.18-1.37) | 1.08(1.01-1.16) | 1.00(0.77-1.29) |
|  |  | ≥70 | 1.22(1.14-1.32) | 1.14(1.06-1.23) | 1.51(1.25-1.82) |
|  | **Sex** | Female | 1.89(1.69-2.13) | 1.49(1.34-1.67) | 1.76(1.07-2.87) |
|  |  | Male | 1.18(1.12-1.23) | 1.08(1.03-1.13) | 1.29(1.11-1.51) |
|  | **Hypertension** | Yes | 1.25(1.18-1.32) | 1.15(1.09-1.22) | 1.27(1.08-1.51) |
|  |  | No | 1.27(1.20-1.36) | 1.17(1.10-1.25) | 1.53(1.18-1.98) |
|  | **Diabetes** | Yes | 1.28(1.18-1.38) | 1.15(1.07-1.24) | 1.75(1.33-2.30) |
|  |  | No | 1.26(1.20-1.33) | 1.18(1.13-1.24) | 1.24(1.05-1.47) |
| **TyG-WC** | **Age** | 40-49 | 1.28(1.14-1.45) | 1.19(1.03-1.36) | 5.32(0.79-35.93) |
|  |  | 50-59 | 1.25(1.16-1.34) | 1.12(1.04-1.20) | 1.93(1.18-3.17) |
|  |  | 60-69 | 1.19(1.12-1.27) | 1.02(0.96-1.09) | 1.05(0.81-1.35) |
|  |  | ≥70 | 1.19(1.11-1.28) | 1.08(1.01-1.16) | 1.30(1.05-1.61) |
|  | **Sex** | Female | 1.26(1.19-1.32) | 1.08(1.02-1.14) | 1.15(0.90-1.48) |
|  |  | Male | 1.16(1.10-1.23) | 1.05(0.99-1.11) | 1.32(1.09-1.61) |
|  | **Hypertension** | Yes | 1.19(1.13-1.25) | 1.07(1.02-1.13) | 1.15(0.96-1.37) |
|  |  | No | 1.25(1.18-1.33) | 1.07(1.01-1.14) | 1.53(1.14-2.04) |
|  | **Diabetes** | Yes | 1.22(1.14-1.31) | 1.07(1.00-1.15) | 1.46(1.13-1.90) |
|  |  | No | 1.25(1.19-1.31) | 1.10(1.05-1.15) | 1.15(0.95-1.40) |
| **TyG-WHtR** | **Age** | 40-49 | 1.37(1.21-1.56) | 1.20(1.04-1.39) | 4.93(0.72-33.69) |
|  |  | 50-59 | 1.28(1.19-1.38) | 1.14(1.05-1.23) | 1.91(1.13-3.23) |
|  |  | 60-69 | 1.18(1.11-1.26) | 1.05(0.99-1.12) | 1.09(0.84-1.41) |
|  |  | ≥70 | 1.17(1.09-1.25) | 1.13(1.05-1.21) | 1.44(1.17-1.78) |
|  | **Sex** | Female | 1.24(1.18-1.31) | 1.12(1.07-1.18) | 1.28(1.01-1.63) |
|  |  | Male | 1.17(1.11-1.24) | 1.07(1.01-1.13) | 1.37(1.11-1.68) |
|  | **Hypertension** | Yes | 1.18(1.12-1.24) | 1.10(1.05-1.16) | 1.18(0.99-1.41) |
|  |  | No | 1.27(1.20-1.35) | 1.11(1.04-1.18) | 1.82(1.35-2.46) |
|  | **Diabetes** | Yes | 1.21(1.13-1.29) | 1.10(1.03-1.18) | 1.53(1.18-1.97) |
|  |  | No | 1.25(1.19-1.31) | 1.14(1.08-1.19) | 1.24(1.02-1.52) |
| **LAP** | **Age** | 40-49 | 1.28(1.06-1.56) | 1.36(1.09-1.70) | 4.43(0.51-38.54) |
|  |  | 50-59 | 1.19(1.07-1.31) | 1.09(1.00-1.19) | 4.39(2.03-9.50) |
|  |  | 60-69 | 1.31(1.18-1.44) | 1.08(0.99-1.19) | 1.10(0.78-1.56) |
|  |  | ≥70 | 1.29(1.15-1.45) | 1.21(1.08-1.35) | 1.76(1.23-2.50) |
|  | **Sex** | Female | 1.32(1.21-1.43) | 1.12(1.04-1.21) | 1.18(0.84-1.67) |
|  |  | Male | 1.23(1.13-1.34) | 1.13(1.04-1.23) | 1.85(1.33-2.58) |
|  | **Hypertension** | Yes | 1.22(1.14-1.31) | 1.10(1.03-1.18) | 1.30(0.99-1.70) |
|  |  | No | 1.35(1.22-1.49) | 1.21(1.10-1.34) | 2.31(1.47-3.65) |
|  | **Diabetes** | Yes | 1.26(1.14-1.38) | 1.18(1.08-1.28) | 1.76(1.19-2.62) |
|  |  | No | 1.28(1.18-1.37) | 1.10(1.02-1.18) | 1.43(1.04-1.97) |
| Adjusted for age, sex, living status, education level, current smoking, alcohol consumption, physical inactivity, hypertension, diabetes, cerebrovascular diseases, heart disease, FBG, LDL-C, TC, TG, HDL-C, BMI. | | | | | |

| **Supplement Table 7.** **Receiver operating characteristic curves of seven surrogate IR indexes for predicting CA, increased CIMT, carotid plaque, or carotid stenosis.** | | | | | | | | | | | |
| --- | --- | --- | --- | --- | --- | --- | --- | --- | --- | --- | --- |
| **Variables** | **CA** | |  | **Increased CIMT** | |  | **Carotid plaque** | |  | **Carotid stenosis** | |
|  | **AUC (95%CI)** | ***P* value** |  | **AUC (95%CI)** | ***P* value** |  | **AUC (95%CI)** | ***P* value** |  | **AUC (95%CI)** | ***P* value** |
| CVAI | 0.638 (0.632-0.645) | Reference |  | 0.619 (0.613-0.626) | Reference |  | 0.625 (0.618-0.632) | Reference |  | 0.628 (0.596-0.660) | Reference |
| TyG | 0.521 (0.514-0.528) | <0.001 |  | 0.512 (0.504-0.519) | <0.001 |  | 0.519 (0.512-0.526) | <0.001 |  | 0.508 (0.475-0.542) | <0.001 |
| TyG-BMI | 0.510 (0.503-0.517) | <0.001 |  | 0.508 (0.501-0.515) | <0.001 |  | 0.503 (0.496-0.510) | <0.001 |  | 0.505 (0.472-0.538) | <0.001 |
| TyG-WC | 0.562 (0.555-0.569) | <0.001 |  | 0.557 (0.550-0.564) | <0.001 |  | 0.548 (0.540-0.555) | <0.001 |  | 0.564 (0.531-0.596) | <0.001 |
| TyG-WHtR | 0.574 (0.567-0.581) | <0.001 |  | 0.563 (0.556-0.570) | <0.001 |  | 0.562 (0.555-0.569) | <0.001 |  | 0.568 (0.536-0.601) | <0.001 |
| VAI | 0.512 (0.505-0.519) | <0.001 |  | 0.511 (0.504-0.518) | <0.001 |  | 0.503 (0.496-0.510) | <0.001 |  | 0.514 (0.480-0.548) | <0.001 |
| LAP | 0.532 (0.525-0.539) | <0.001 |  | 0.531 (0.524-0.538) | <0.001 |  | 0.518 (0.511-0.526) | <0.001 |  | 0.507 (0.474-0.540) | <0.001 |

Abbreviations: IR, insulin resistance; CA, carotid atherosclerosis; CIMT, carotid intima-media thickness; AUC, area under the curve; CVAI, the Chinese visceral adiposity index; TyG, triglyceride‐glucose index; BMI, body mass index; WC, waist circumferences; WHtR, waist-to-height-ratio; VAI, the visceral adiposity index; LAP, lipid accumulation product.

| **Supplement Table 8.** **Receiver operating characteristic curves of seven surrogate IR indexes for predicting CA, increased CIMT, carotid plaque, or carotid stenosis among individuals without dyslipidemia.** | | | | | | | | | | | |
| --- | --- | --- | --- | --- | --- | --- | --- | --- | --- | --- | --- |
| **Variables** | **CA** | |  | **Increased CIMT** | |  | **Carotid plaque** | |  | **Carotid stenosis** | |
|  | **AUC (95%CI)** | ***P* value** |  | **AUC (95%CI)** | ***P* value** |  | **AUC (95%CI)** | ***P* value** |  | **AUC (95%CI)** | ***P* value** |
| CVAI | 0.655 (0.646-0.664) | Reference |  | 0.633 (0.623-0.642) | Reference |  | 0.644 (0.634-0.654) | Reference |  | 0.637 (0.586-0.688) | Reference |
| TyG | 0.517 (0.507-0.526) | <0.001 |  | 0.505 (0.495-0.515) | <0.001 |  | 0.517 (0.508-0.527) | <0.001 |  | 0.519 (0.468-0.569) | <0.001 |
| TyG-BMI | 0.501 (0.491-0.510) | <0.001 |  | 0.501 (0.491-0.511) | <0.001 |  | 0.504 (0.494-0.514) | <0.001 |  | 0.522 (0.471-0.572) | <0.001 |
| TyG-WC | 0.562 (0.553-0.572) | <0.001 |  | 0.559 (0.549-0.569) | <0.001 |  | 0.551 (0.541-0.561) | <0.001 |  | 0.556 (0.508-0.604) | <0.001 |
| TyG-WHtR | 0.579 (0.569-0.588) | <0.001 |  | 0.570 (0.560-0.580) | <0.001 |  | 0.569 (0.559-0.579) | <0.001 |  | 0.574 (0.524-0.624) | <0.001 |
| VAI | 0.507 (0.497-0.516) | <0.001 |  | 0.505 (0.495-0.515) | <0.001 |  | 0.505 (0.495-0.515) | <0.001 |  | 0.575 (0.527-0.624) | <0.001 |
| LAP | 0.523 (0.514-0.533) | <0.001 |  | 0.527 (0.517-0.537) | <0.001 |  | 0.510 (0.500-0.520) | <0.001 |  | 0.527 (0.479-0.575) | <0.001 |

Abbreviations: IR, insulin resistance; CA, carotid atherosclerosis; CIMT, carotid intima-media thickness; AUC, area under the curve; CVAI, the Chinese visceral adiposity index; TyG, triglyceride‐glucose index; BMI, body mass index; WC, waist circumferences; WHtR, waist-to-height-ratio; VAI, the visceral adiposity index; LAP, lipid accumulation product.
